# Changes in Apoptotic Pathways in MOLM-13 Cell Lines after Induction of Resistance to Hypomethylating Agents

**DOI:** 10.3390/ijms22042076

**Published:** 2021-02-19

**Authors:** Ľuboš Janotka, Lucia Messingerová, Kristína Šimoničová, Helena Kavcová, Katarína Elefantová, Zdena Sulová, Albert Breier

**Affiliations:** 1 Institute of Molecular Physiology and Genetics, Centre of Biosciences, Slovak Academy of Sciences, Dúbravská cesta 9, 840 05 Bratislava, Slovakia; lubos.janotka@savba.sk (Ľ.J.); kristina.simonicova@savba.sk (K.Š.); helena.kavcova@savba.sk (H.K.); 2 Institute of Biochemistry and Microbiology, Faculty of Chemical and Food Technology, Slovak University of Technology in Bratislava, Radlinského 9, 812 37 Bratislava, Slovakia; katarina.elefantova@stuba.sk

**Keywords:** AML cell drug resistance, 5-aza-2′-deoxycytidine, 5-azacytidine, intrinsic and extrinsic pathways of apoptosis, promoter methylation, NF-κB pathway

## Abstract

We established the following two variants of the MOLM-13 human acute myeloid leukemia (AML) cell line: (i) MOLM-13/DAC cells are resistant to 5-aza-2′-deoxycytidine (DAC), and (ii) MOLM-13/AZA are resistant to 5-azacytidine (AZA). Both cell variants were obtained through a six-month selection/adaptation procedure with a stepwise increase in the concentration of either DAC or AZA. MOLM-13/DAC cells are resistant to DAC, and MOLM-13/AZA cells are resistant to AZA (approximately 50-fold and 20-fold, respectively), but cross-resistance of MOLM-13/DAC to AZA and of MOLM-13/AZA to DAC was not detected. By measuring the cell retention of fluorescein-linked annexin V and propidium iodide, we showed an apoptotic mode of death for MOLM-13 cells after treatment with either DAC or AZA, for MOLM-13/DAC cells after treatment with AZA, and for MOLM-13/AZA cells after treatment with DAC. When cells progressed to apoptosis, via JC-1 (5,5′,6,6′-tetrachloro-1,1′,3,3′-tetraethyl-imidacarbocyanine iodide) assay, we detected a reduction in the mitochondrial membrane potential. Furthermore, we characterized promoter methylation levels for some genes encoding proteins regulating apoptosis and the relation of this methylation to the expression of the respective genes. In addition, we focused on determining the expression levels and activity of intrinsic and extrinsic apoptosis pathway proteins.

## 1. Introduction

Myelodysplastic syndrome (MDS) represents a heterogeneous group of clonal myeloid neoplasms characterized by ineffective hematopoiesis. Patients with high-risk MDS may progress to acute myeloid leukemia (AML) over time [1]. One common feature of these diseases is the enhanced number of aberrantly differentiated myeloblasts in peripheral blood [2]. For patients who are not eligible for stem cell transplantation, hypomethylating agent (HMA) therapy is an effective treatment option. Two HMA agents, 5-azacytidine (AZA) and 5-aza-2′-deoxycytidine (DAC), have been approved for the treatment of high-risk MDS patients [3]. Both AZA and DAC, currently known as hypomethylating agents, were first synthesized and studied in Czechoslovakia as antimetabolites [4,5]. While AZA contains ribose and is preferentially incorporated into RNA (65–90%) and into DNA at a much lower level (10–35%), DAC contains 2-deoxyribose and is incorporated exclusively into DNA [6,7]. The mechanism of AZA and DAC action has not been fully elucidated. In addition to direct effects as cytosine derivatives incorporated into DNA when demethylation is induced, these substances may alter the immunological response to the presence of neoplastically transformed cells or induce several cytotoxic effects, including disruption of nucleic acid and protein metabolism, which leads to apoptosis [8,9,10]. These responses make HMA therapy response prediction difficult, and until now, specific molecular assays for distinguishing responders from nonresponders were unavailable [6,11]. The complete response rate to AZA and DAC varies from 7 to 35% [12,13,14,15]. The outcome for patients after HMA failure is poor [16,17]. Mechanisms of resistance to these agents in AML and MDS patients are still not fully understood but may include changes in metabolic pathways, regulation of apoptosis, cell cycle progression or mutation-specific genes [18,19,20,21,22].

Several apoptosis-associated genes, including *TNFRSF25* (tumor necrosis factor receptor superfamily member 25), were found to be methylated in HMC-1.2 cells (established from a patient with mast cell leukemia) but not in normal bone marrow leukocytes, suggesting that these genes are aberrantly hypermethylated in blood neoplasia [23]. The product of the *TNFRSF25* gene, known as death receptor 3 (DR3), is a membrane protein consisting of 417 amino acids with a molecular weight of approximately 45 kDa. Alteration of the NF-κB regulatory pathway downstream of DR3 leads to subtle changes in the antiapoptotic/proapoptotic balance, and the prevalence of extrinsic apoptotic stimuli via activation of caspase 8 is evident [24]. Differences between the two apoptotic pathways are thought to be associated with signaling upon recruitment of the TNFR-associated death domain (TRADD) or Fas-associated death domain (FADD) protein to the cytosolic tail of DR3 [25].

In a previous paper, we prepared cell variants resistant to AZA using human AML cells MOLM-13 and SKM-1, which overexpress P-glycoprotein (P-gp) and are cross-resistant to P-gp substrates [26,27]. In the current paper, we describe the preparation of MOLM-13 cell variants specifically resistant to either AZA or DAC. In these cell variants, we have studied the alteration of several molecular features that may be critical for decreased cell sensitivity to HMAs.

## 2. Results

### 2.1. Introduction of AZA- and DAC-Resistant MOLM-13 Cell Variants

**Figure 1:**
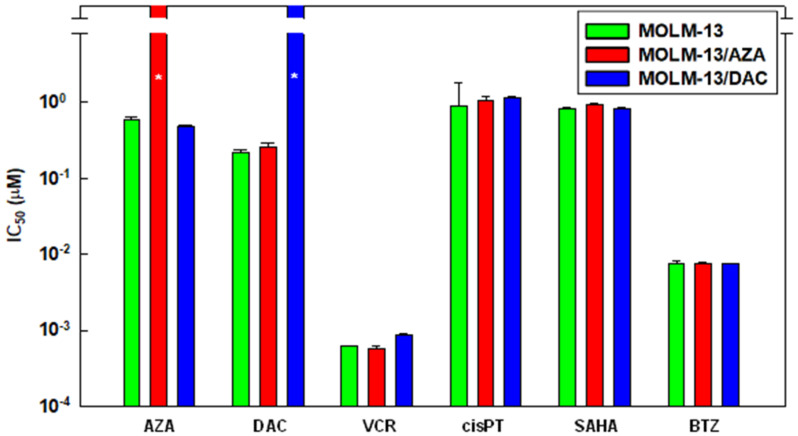
Cell sensitivities to 5-azacytidine (AZA), 5-aza-2′-deoxycytidine (DAC), vincristine (VCR), suberoylanilide hydroxamic acid (SAHA) and bortezomib (BTZ) are expressed as the median inhibitory concentration (IC_50_). The IC_50_ was computed by nonlinear regression according to Equation 1 using SigmaPlot for Windows (Version 8.02, Systat Software GmbH, Erkrath, Germany). The data represent computed value ± standard error with 30 degrees of freedom. * = Even at a concentration of 10 μM, neither AZA nor DAC showed a half-maximal inhibitory effect on the cells.

The cell variants MOLM-13/AZA and MOLM-13/DAC were obtained by culturing original MOLM-13 cells for half a year in stepwise increasing concentrations of either AZA or DAC. During this process, the expression of ABC (ATP-binding cassette) transporters was monitored. Finally, we obtained the following two cell variants: one variant resistant to AZA—MOLM-13/AZA, and one variant resistant to DAC—MOLM-13/DAC. The resistance ratios were higher than 20-fold and 50-fold, respectively. However, no cross-resistance of either MOLM 13/AZA to DAC or MOLM-13/DAC to AZA was observed (Figure 1). Moreover, we did not observe a considerable decrease in the cell sensitivity of either MOLM-13 cell variant to the following drugs: (i) vincristine (VCR) a P-glycoprotein substrate that is used in the treatment of various malignancies, including acute leukemia; (ii) cisplatin (cisPt), a typical alkylating agent used in cancer therapy; (iii) bortezomib (BTZ; a proteasome inhibitor used in the treatment of multiple myeloma and mantle cell lymphoma); (iv) vorinostat (also known as suberoylanilide hydroxamic acid– SAHA, an inhibitor of histone deacetylase used for the epigenetic therapy of patients with cutaneous T-cell lymphoma.

**Figure 2:**
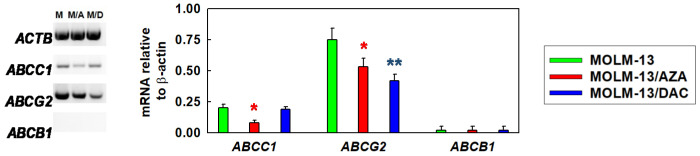
Expression of ABC (ATP-binding cassette) transporters in MOLM-13, MOLM-13/AZA, and MOLM-13/DAC cells as determined by RT-PCR. Electrophoretic gels were quantified by densitometry and summarized in column plots. The gel is representative of three independent measurements. The data in the column plot represent the mean ± SD of three independent measurements. Significance: * = *p* <0.05; ** = *p* <0.02.

Three membrane transporters, ABCB1 (P-glycoprotein), ABCC1 (multidrug resistance-associated protein 1 [28]) and ABCG2 (breast cancer resistant protein [29]), often play a role in the development of a multidrug resistant phenotype [9]. However, no increase in the expression of these transporters at the mRNA level in the MOLM-13 cell variants resistant to AZA or DAC was observed (Figure 2).

In contrast, a slight but statistically significant decrease in the mRNA encoding ABCG2 was observed in both the MOLM-13/AZA and MOLM-13/DAC cell variants compared with the parental MOLM-13 cells. The amount of mRNA encoding the ABCC1 transporter was significantly decreased only in the MOLM-13/AZA cells, not in MOLM-13/DAC cells. Only negligible (if any) amounts of mRNA encoding *ABCB1* transporters were observed in the three types of MOLM-13 cell types (Figure 2). In comparison, MOLM-13 cells selected for VCR resistance that were also resistant to other P-gp substrates expressed large amounts of P-gp [30].

### 2.2. Effect of AZA and DAC on Apoptosis/Necrosis Progression in the Sensitive MOLM-13 Cell Line and the Resistant MOLM-13/AZA and MOLM-13/DAC Cell Variants

**Figure 3:**
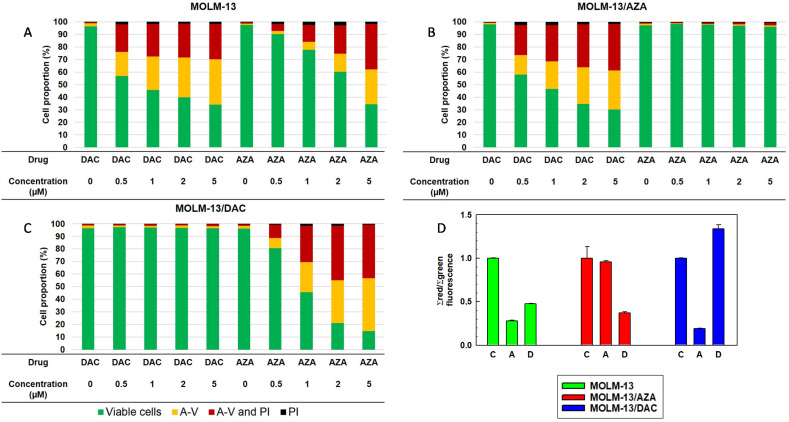
Flow cytometry characterization of viability (using double staining with FITC-linked annexin V (FAV) and propidium iodide (PI); (**A**–**C**) and mitochondrial membrane potential (using the red and green staining of JC-1 (5,5′,6,6′-tetrachloro-1,1′,3,3′-tetraethyl-imidacarbocyanine iodide) [31]; (**D**) of all three MOLM-13 cell types. Panel A—MOLM-13, B—MOLM-13/AZA and C—MOLM-13/DAC cells were incubated for 72 h in growth medium in the absence or presence of AZA or DAC (0.0, 0.5, 1.0, 2.0, and 5.0 µM) in a CO_2_ incubator. The cells were treated with AZA or DAC repeatedly every 24 h. After 72 h of cultivation, apoptosis/necrosis progression was assayed using an FAV/PI kit. Specific cell fluorescence was measured by fluorescence flow cytometry. Typical dot blot samples are shown in Supplementary file Figure S1A. The data are expressed as the median of the florescence intensity of the FAV- or PI-labeled cells and represent means from three independent values. The SD of these measurements never exceeded 10%. Viable cells—unstained cells; apoptotic cells (A-V) stained with FAV; necrotic cells (PI) stained with PI; cells in late apoptosis/necrosis (A-V and PI) stained with both FAV and PI. Panel D: Detection of mitochondrial membrane potential with JC-1 in all three MOLM-13 cell types untreated (**C**) or treated with AZA (**A**) and DAC (**D**). Ratios of red/green fluorescence were used as measures of mitochondrial membrane potential (MMP). The data were determined using the sum of the JC-1 green and red fluorescence intensities obtained from flow cytometry and represent the mean ± SD of three independent measurements. These measurements were obtained using a protocol described elsewhere [31]. To prove the relationship between JC-1 red and green fluorescence and MMP, cells with depolarized mitochondria due to the application of carbonyl cyanide m-chlorophenylhydrazone (CCCP), a mitochondrial membrane potential uncoupler, were assessed (Supplementary file Figure S1B) according to the recommendation of the BD Accuri Cytometer (BD Biosciences, San Jose, CA, USA) protocol [32].

After incubation of MOLM-13, MOLM-13/AZA and MOLM-13/DAC cells with AZA and DAC in the concentration range of 0–5 μΜ, we observed significant changes in apoptosis/necrosis progression using double labeling of cells with FITC-linked annexin V (FAV) and propidium iodide (PI). Both AZA and DAC induced a concentration-dependent decrease in viable parental sensitive cells. Incubation of the parental cells with 1 µM DAC caused the viability to decrease to one-half, and incubation with 5 µM DAC reduced cell viability to one-third (Figure 3A). Incubation with 1 µM AZA decreased cell viability to 77%, and with 5 µM, it dropped to less than 40%. We proved that either MOLM 13/AZA and MOLM-13/DAC were able to grow and survive during 72 h of culture in medium containing either AZA or DAC (5 µM), respectively (Figure 3B,C). On the other hand, the sensitivity of MOLM-13/AZA to DAC and MOLM-13/DAC to AZA remained. Both AZA-induced cell death in MOLM-13 and MOLM-13/DAC cell variants, or DAC-induced cell death in MOLM-13 and MOLM-13/AZA cell variants are predominantly driven by apoptosis. This result was shown by the predominant labeling of FAV alone and FAV together with PI, indicating apoptosis, but only a small proportion of cells were labeled with PI only (Figure 3A). However, DAC induced apoptosis (indicated by staining with either FAV alone or FAV and PI together) in MOLM-13/AZA cells (Figure 3B), and similarly, AZA induced cell death in MOLM-13/DAC cells (Figure 3C). These results indicated the unchanged sensitivity of the MOLM-13/DAC cells to AZA and the MOLM-13/AZA cells to DAC. During apoptosis progression, a decrease in the mitochondrial membrane potential (MMP) is evident in overall cell death mechanisms. Therefore, we detected the effect of AZA and DAC on the MMP in all three MOLM-13 cell types using JC-1 (5,5′,6,6′-tetrachloro-1,1′,3,3′-tetraethyl-imidacarbocyanine iodide) red/green double staining [31]. While AZA induced a reduction in the MMP in MOLM-13 and MOLM-13/DAC cells, DAC induced a reduction in the MMP in MOLM-13 and MOLM- 13/AZA cells (Figure 3D). However, the MMP remained unchanged in the MOLM-13/AZA cells after treatment with AZA and the MOLM-13/DAC cells after treatment with DAC.

**Figure 4:**
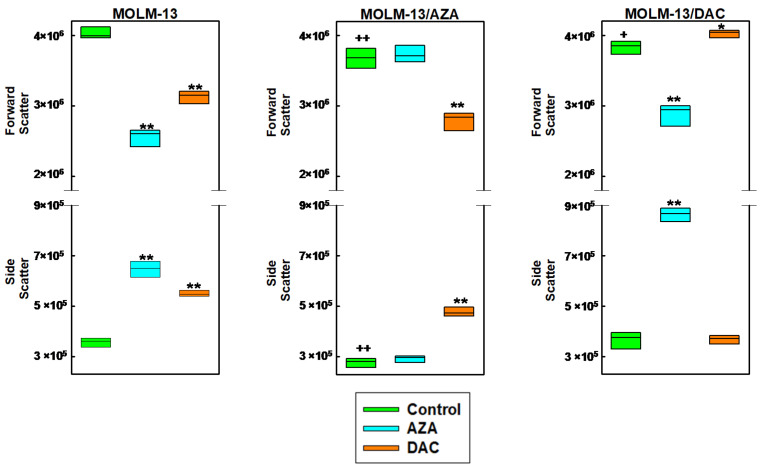
Box plots showing the forward and side scatter of MOLM-13, MOLM-13/DAC, and MOLM-13/AZA cells cultivated in the presence or absence of AZA or DAC (1 μM). The boxes were generated by SigmaPlot for Windows (Version 8.02, Systat Software GmbH, Erkrath, Germany) based on three independent measurements. Statistical significance: ^++^ and ^+^ = values differ from the data obtained for the MOLM-13 cells at the level of *p* <0.02 and *p* <0.05, respectively; ** and * = values differ from the data obtained for the respective cell types in the absence of AZA and DAC at the level *p* <0.01 and *p* <0.02, respectively. The top and bottom of each box represent the 5th and 95th percentiles, respectively, and the horizontal line in the box represents the median value.

The process of apoptosis is associated with typical morphological manifestations such as cell shrinkage and the formation of intracellular apoptotic granules [33,34]. Using fluorescence cytometry, we measured changes in the size and granularity of all three MOLM-13 cell types. Forward and side scatter are measures of cell size and granularity, respectively [35,36]. For these experiments, we were inspired by the findings of Wlodkowic et al. [37], who showed that apoptotic cells have reduced forward scatter, while their side scatter is enhanced. MOLM 13/AZA cells showed a significant decrease in cell granularity and size (lower side and forward scatter) compared to MOLM-13 cells (Figure 4).

In a comparison with the MOLM-13/DAC and MOLM-13 cell variants, only forward scatter was reduced with no changes in side scatter (Figure 4). Both AZA and DAC induced significant MOLM-13 cell size reductions (decreased forward scatter) and increased cell granularity (increased side scatter), as shown in Figure 4, which is consistent with the induction of apoptosis, as detected by FAV/PI after the treatment of MOLM-13 cells with either AZA or DAC (Figure 3). Significant increases in side scatter and decreases in forward scatter were also detected when the MOLM-13/AZA cells were treated with DAC or the MOLM-13/DAC cells were treated with AZA. However, no changes in side scatter or forward scatter were observed when the MOLM-13/AZA cells were treated with AZA or the MOLM-13/DAC were treated with DAC (Figure 4).

For further evaluation, we excluded damaged cells that were stained with FAV or PI from the evaluation and measured the forward and side scatter. Although only live cells were counted, the decrease in both scatter patterns for the MOLM-13/AZA cells compared to those of the MOLM-13 cells remained (Supplementary file Figure S2), and neither the forward nor side scatter changed for the MOLM-13/DAC cells. Culturing with AZA and DAC also reduced forward scatter and increased side scatter in the MOLM-13 cells. Culturing MOLM-13/DAC in the presence of AZA and MOLM-13/AZA in the presence of DAC resulted in an increase in side scatter in the surviving cells without altering the forward scatter. DAC treatment of the MOLM-13/DAC cells and AZA treatment of MOLM-13/AZA cells did not induce significant changes in these parameters (Supplementary file Figure S2).

### 2.3. Methylation Status of Several Genes Involved in the Regulation of Apoptosis of MOLM-13, MOLM-13/AZA and MOLM-13/DAC Cells

**Figure 5:**
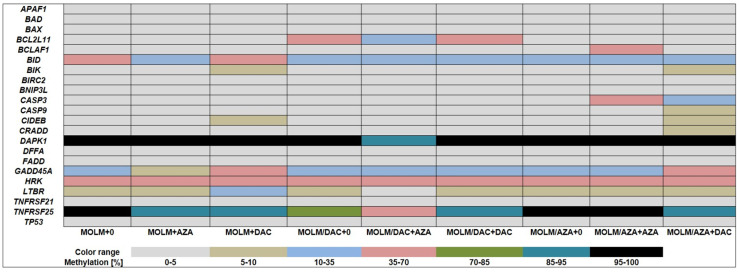
DNA methylation level in apoptosis-associated genes in the sensitive MOLM-13 cell line and resistant MOLM-13/AZA and MOLM-13/DAC variant cell sublines. The Cells were incubated for 72 h in growth medium in the absence (0) or presence of AZA (1 µM) or DAC (1 µM) in a CO_2_ incubator. The cells were treated with AZA or DAC every 24 h.

We measured the promoter methylation of 22 genes associated with the regulation of apoptosis (shown in Table 1) with an EpiTect^®^ Methyl II Signature PCR Array Kit (Qiagen, Hilden, Germany) according to the manufacturer’s protocol [38]. Methylation status was determined after 72 h of incubation of all three cell types in the absence or presence of either DAC or AZA (Figure 5).

We observed several changes in methylation status in these three cell types, possibly as a result of the HMAs present in the cultivation medium. Comparing resistant cell variants to the parental cell line MOLM-13, we observed a decrease in *BID* methylation in both the MOLM-13/AZA and MOLM-13/DAC variant cells. In addition, MOLM-13/DAC cells had a more methylated *BCL2L11* gene and a less methylated *TNFRSF25* gene. Treatment of these cells with HMAs led to changes in methylation levels.

Some of these changes seem to correlate with the response of these cells to the drugs. We detected a slight increase in methylation in *CIDEB* and *BIK* in response to DAC, with a more significant increase in *GADD45A* in response to DAC and a decrease in methylation of *TNFRS25* promoter was detected in response to both HMAs. We did not observe intrinsic or HMA-stimulated changes in the methylation of 10 genes in the cell types in cultivation medium (characterized by the full gray row in Figure 5). Surprisingly, an increase in methylation of some genes (*BIK*, *CASP3*, *CIDEB*, *GADD45A*, *LTBR*, *BCLAF1*, *CASP9*, and *CRADD*) was induced by treatment with HMAs.

### 2.4. Relative Expression of CASP3, DAPK1, GADD45A, TNFRSF25, BCL2L11, BCLAF1, BID, and BIK in Sensitive and Resistant Cell Lines and Its Relationship to the Methylation Status of the Respective Promoters

**Figure 6:**
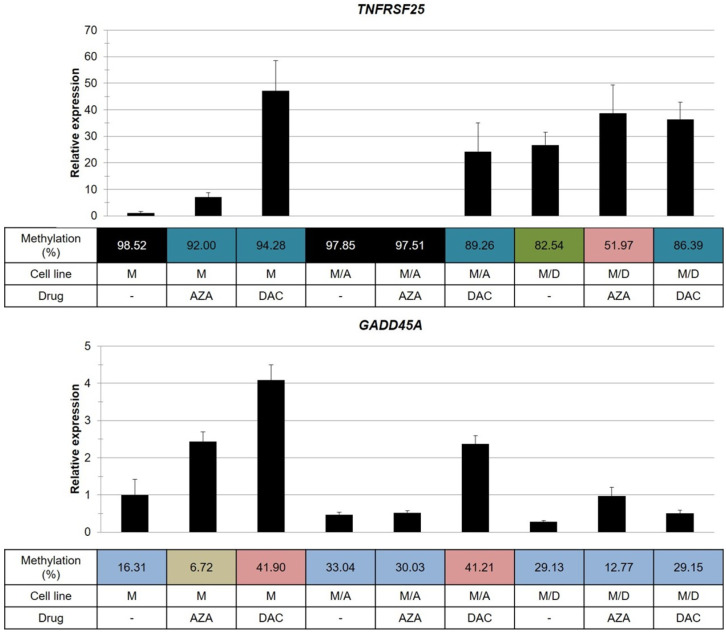
Changes in the relative expression and methylation of *GADD45A* and *TNFRSF25* in sensitive and resistant cell lines. M—MOLM-13-sensitive cell line; M/A—MOLM-13/AZA, AZA-resistant subline; M/D—MOLM-13/DAC, DAC-resistant subline. The cells were incubated for 72 h in growth medium in the absence or presence of AZA (1 µM) or DAC (1 µM) in a CO_2_ incubator. The cells were treated every 24 h with either AZA or DAC. *ACTB* was used as an internal control. The data are expressed as the mean ± SD of three independent measurements.

We measured the relative expression of *CASP3*, *DAPK1*, *GADD45A*, *TNFRSF25*, *BCL2L11*, *BCLAF1*, *BID* and *BIK* at the mRNA level by qRT-PCR after 72 h of cell cultivation with either DAC or AZA. We intended to study the connection between promoter methylation of these genes and their transcription levels (Figure 6 and Supplementary files Figure S3).

The relative expression levels of *CASP3*, *BCL2L11*, *BCLAF1*, *BID*, *GADD45A* and *BIK* were not correlated with the promoter methylation of these genes. This result indicated that changes in methylation of the gene promoter under our conditions had no meaningful influence on their expression levels. We detected lower expression of *BID* (proapoptotic gene [39,40]) in both resistant variants compared to the parental cell line (Supplementary file Figure S3).

While there was no change in the expression of *BID* in the MOLM-13/AZA cells treated with HMAs, we observed the upregulation of these genes in MOLM-13/DAC cells, treated with either HMA. On the other hand, we observed significant downregulation of *BIK* (proapoptotic gene [40]) in the MOLM-13/DAC cells compared to the level in both the MOLM-13 and MOLM-13/AZA cells (Supplementary file Figure S3). There was no significant change in transcription of this gene when the MOLM-13/DAC cells were treated with the HMAs (Supplementary file Figure S3). In contrast, both HMAs induced significant elevation of *BIK* in the MOLM-13/AZA cells. Furthermore, we observed the upregulation of executive caspase *CASP3* [41] and *BCL2L11* (known BIM proapoptotic genes [40]) in the MOLM-13/AZA cells treated with DAC (Supplementary file Figure S3).

A slight decrease in methylation of the *DAPK1* promoter was observed only in one sample cell line, the MOLM 13/DAC variant treated with AZA, while the methylation of the other two cell types, in which the promoter was fully methylated, remained unchanged. Even a small decrease in methylation seems to promote the expression of the *DAPK1* gene, as indicated by a comparison of the AZA-treated MOLM-13/DAC cells with the DAC-treated or untreated MOLM-13/DAC cells. Except for these two cell samples, very little or no DAPK1 mRNA was detected. However, the expression of *DAPK1* was observed in samples with fully methylated promoters (MOLM-13 and MOLM-13/AZA cells treated with DAC). These results suggest that other mechanisms of transcription regulation may overcome the effect of methylation. *DAPK1* is a proapoptotic gene [42], and it seems that its high relative upregulation is correlated with the effect of DAC in our cell models (Supplementary file Figure S3).

In the *TNFRSF25* gene, a fully methylated promoter was observed in three cell samples: The untreated MOLM-13 and MOLM-13/AZA cells and the MOLM-13/AZA cells treated with AZA (Figure 6). In all three cases, very little or no *TNFRSF25* mRNA was detected. Furthermore, we detected a decrease in the methylation of this promoter, which was caused by the effect of the HMAs. It seems that a slight decrease in promoter methylation allows transcription of *TNFRSF25*. We found an approximately seven-fold increase in the expression of this gene in AZA-treated MOLM-13 cells and an approximately 50-fold increase in DAC-treated MOLM-13 cells. MOLM-13/AZA cells show completely suppressed expression of the *TNFRSF25* gene and methylation of the promoter of this gene greater than 95% (Figure 6). This pattern is consistent even when the cell variants have been treated with AZA. However, treatment with DAC suppressed the methylation of the promoter of this gene to be less than 90%, and its detectable expression was also observed. We observed a considerable reduction in the methylation of the *TNFRSF25* gene promoter (below 85%) in MOLM-13/DAC cells compared to the parental cells, which is related to the increased expression of this gene (Figure 6). AZA, but not DAC, induced a further decrease in the promoter methylation of this gene, to less than 55%, but this did not lead to a significant increase in the level of its mRNA.

Increased *GADD45A* expression was observed in MOLM-13 cells after both AZA and DAC treatment, in MOLM-13/AZA cells after DAC treatment, and in MOLM-13/DAC cells after AZA treatment. Interestingly, these cases of upregulation were detected after DAC treatment of the MOLM-13 and MOLM-13/AZA cells, although the methylation of the *GADD45A* promoter was found to be increased (Figure 6).

The *TNFRSF25* and *GADD45A* genes show different behaviors. In the former gene, transcription follows a decrease in the methylation of its promoter, whereas in the latter gene, this phenomenon did not occur.

### 2.5. Changes in the Protein Levels of BCL2 and BAX in the MOLM-13, MOLM-13/DAC, MOLM-13/AZA Cells

**Figure 7:**
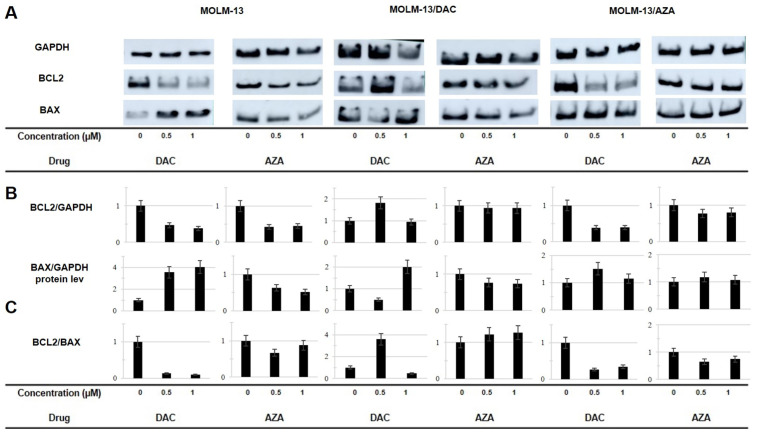
(**A**): Levels of BCL2 and BAX proteins in sensitive and resistant cell lines as determined by Western blot analysis. The cells were incubated for 72 h in growth medium in the absence or presence of AZA or DAC in a CO_2_ incubator. The results of 24 and 48 h of incubation are shown in Supplementary file Figures S4 and S5. AZA or DAC was added to the cultivation medium every 24 h. The level of GAPDH was used as an internal control. The data are representative of three independent measurements. (**B**): The optical densities of the protein bands were quantified by densitometry and are summarized in the bar plots. The data are expressed as the mean ± SD of three independent measurements. (**C**): Ratios of BCL2/BAX proteins (as a measure of the internal pathway of apoptosis [44]) are indicated by the data in panel C.

To characterize the involvement of the intrinsic apoptosis pathway in HMA-induced cell death, the expression of BCL2 and BAX, typical antiapoptotic and proapoptotic proteins, respectively, was analyzed. BCL2 and BAX are the best-characterized members of the BCL2 gene family. BAX is critical for the permeabilization of the outer mitochondrial membrane in the internal apoptosis pathway, and BCL2 is a BAX antagonist [43]. The expression of both genes at the protein level was detected by Western blotting after cell culture in the presence or absence of DAC and AZA for 72 h (Figure 7) as well as for 24 or 48 h (Supplementary files Figures S4 and S5).

In parental MOLM-13 cells, the downregulation of BCL2 was detected after cultivation with both DAC and AZA. The greatest difference in the effect of DAC and AZA in the MOLM-13 cells was a four-fold upregulation of BAX after cultivation with DAC a downregulation of this protein after cultivation with AZA (Figure 7).

In the MOLM-13/DAC cells, upregulation of BAX was also observed after cultivation with DAC (1 μM), but the protein level of BCL2 was not decreased. In these cells, there was no change in the protein level of BCL2 and only a slight downregulation of BAX after treatment with AZA.

In the AZA-resistant MOLM-13/AZA cells, a decrease in the protein level of BCL2 was observed after treatment with DAC. This downregulation was very similar to the decrease observed in the parental MOLM-13 cells after DAC treatment (Figure 7). Further, upregulation of BAX was also observed after DAC treatment (0.5 μM). On the other hand, after cultivation of the MOLM-13/AZA cells with AZA, there was only a slight change in BCL2, and no change was observed in BAX expression.

DAC induced the strongest reduction in the BCL2/BAX ratio in the MOLM-13 and MOLM-13/AZA cells. In the MOLM-13/DAC cells, the effect was the opposite, with this ratio increased approximately 3.5-fold with 0.5 μM DAC but decreased to one-half this amount with 1.0 μM DAC, compared to the untreated control in both cases.

### 2.6. Expression of the REL, RELA, RELB, NFKB1 and NFKB2 Genes in the Sensitive and Resistant Cell Lines

**Figure 8:**
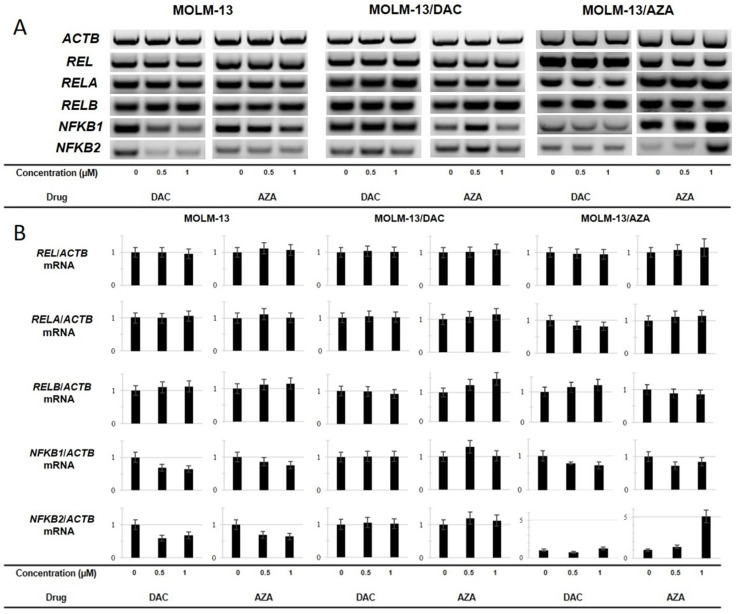
(**A**): Changes in the relative expression of the *REL*, *RELA*, *RELB*, *NFKB1* and *NFKB2* genes in sensitive and resistant cell lines as determined by RT-PCR. The cells were incubated for 72 h in growth medium in the absence or presence of AZA or DAC in a CO_2_ incubator. The data for 24 and 48 h of incubation are shown in the Supplementary files (Figures S6 and S7 in the Supplementary data). The cells were treated every 24 h with AZA or DAC. *ACTB* was used as an internal control. (**B**): The optical densities of the PCR product bands were quantified and are summarized in bar plots. The data are expressed as the mean ± SD of three independent measurements.

We measured the mRNA levels of members of the NF-κB signaling pathway to further analyze the response of MOLM-13 cells and our HMA-resistant cell variants to treatment with HMAs. We analyzed the mRNA levels of the *REL*, *RELA*, *RELB*, *NFKB1* and *NFKB2* genes by RT-PCR. In these experiments, the *ACTB* gene was used as an internal control (Figure 8). There was no change in the mRNA levels of the *REL*, *RELA*, or *RELB* genes in the MOLM-13 cells after treatment with HMAs. In the sensitive cell line, the most significant change was the downregulation of *NFKB1* and *NFKB2* genes after treatment with AZA and DAC. DAC seems to have the same effect on the transcription of both of these genes, while treatment with AZA led to less significant downregulation of *NFKB1*.

In the DAC-resistant cell variant, MOLM-13/DAC cell line, neither the *NFKB1* nor the *NFKB2* gene was downregulated. Furthermore, no changes in the expression of any member of the NF-κB signaling pathway were observed in the MOLM-13/DAC cells after cultivation with DAC. On the other hand, in this cell variant, upregulation of the *RELB* gene was detected after treatment with AZA. Less significant upregulation of the *RELB* gene was also detected in the MOLM-13/AZA cells after treatment with DAC. More interestingly, in the MOLM-13/AZA cells treated with AZA, a significant five-fold change in the level of *NFKB2* mRNA was detected, which was the most significant change in response to a HMA when the parental MOLM-13 cells and resistant variant cells were compared.

### 2.7. AZA- and DAC-Induced Activation of Caspase 3/7, 8, and 9 in All Three Types of MOLM-13 Cells

Caspase activities were determined using a Cell MeterTM Multiplexing Caspase 3/7, 8 and 9 Activity Assay Kit (AAT Bioquest, Inc. Sunnyvale, CA through Scintila, s.r.o. Jihlava, Czech Republic) based on the release of fluorescent labels from their conjugates with oligopeptides as specific substrates: ProRed labeled tetrapeptid aspartate-glutamate-valine-aspartate (DEVD-ProRed™)—caspase-3/7 substrate, emitting red fluorescence; isoleucin-glutamate-threonine-aspartate labeled with Rhodamine 110 (IETD-R110)—caspase-8 substrate, emitting green fluorescence; leucine-glutamate-histidine-aspatate labeled with 7-Amino-4-Methylcoumarin (LEHD-AMC)—caspase-9 substrate, emitting blue fluorescence [45]. When the resistant variant MOLM-13/DAC or MOLM-13/AZA cells were cultured in the absence of HMA immediately prior to harvest, we did not find significant differences in caspase activity compared with the parental MOLM-13 cells, as the levels of probability for even marginal significance (*p* < 0.10) were not reached (Table 2).

**Figure 9:**
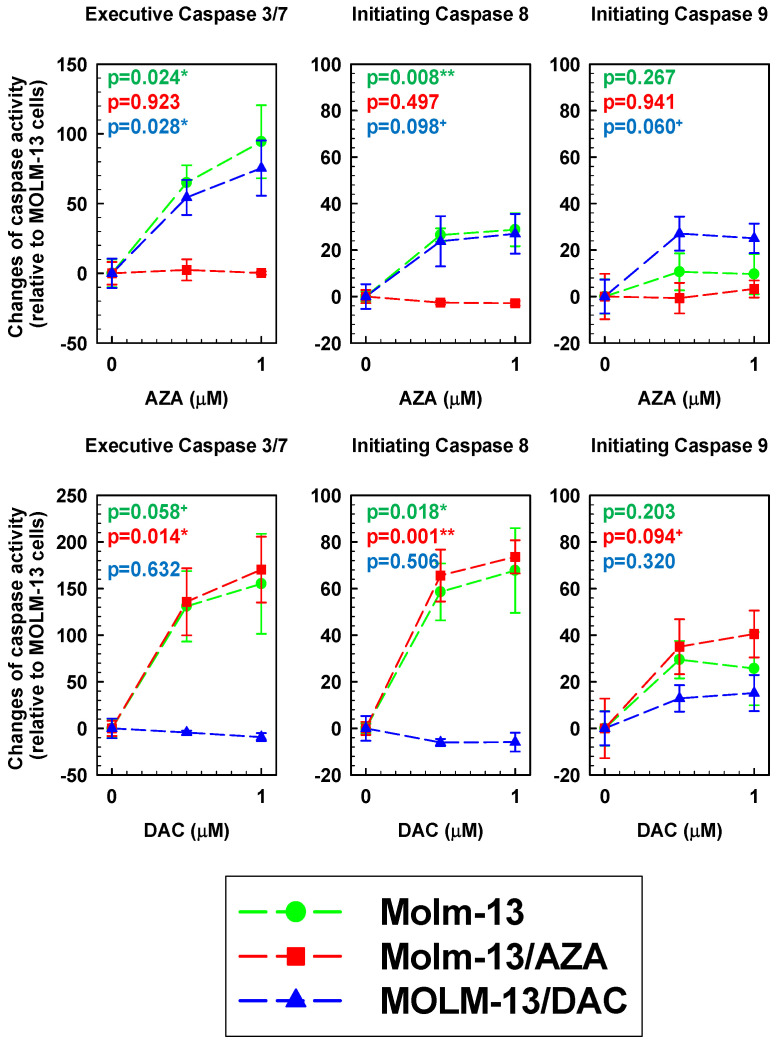
Activity of executive caspase 3/7 and initiating caspase 8 and 9 in the MOLM-13, MOLM-13/AZA and MOLM-13/DAC cells after 72 h in culture in the absence or presence of either AZA or DAC at given concentrations. The data (mean ± SD) of three independent measurements are expressed as percentages representing the difference between the respective value and the value obtained for the same cell variant cultured in the absence of both hypomethylating agent (HMAs). The level of probability (calculated using ANOVA one-way analysis of variance); *p* < 0.01 (**), *p* < 0.05 (*) and *p* < 0.1 (+) were considered highly significant, moderately significant and marginally significant, respectively.

DAC and AZA at concentrations of 0.5 and 1.0 μM induced different changes in caspase activity after 72 h of incubation with MOLM-13, MOLM-13/AZA and MOLM-13/DAC cells (Figure 9). These changes were obtained in the presence of either AZA or DAC and are expressed as a percentage of the difference between the respective value and the value obtained for the same cell variants but cultured in the absence of HMAs. AZA caused significant activation of both initiating caspase 8 and executive caspase 3/7 without significant changes in the activity of initiating caspase 9 in the MOLM-13 cells.

However, DAC induced significant activation of caspase 8, but only a marginally significant increase in caspase 3/7 (0.05 < *p* < 0.10). Activation of caspase 8 and 3/7 in the MOLM-13/AZA cell variant was observed after treatment with DAC but not with AZA. In addition, DAC treatment of this cell variant also caused activation of caspase 9 to a level that met the criterion for marginal significance (Figure 9). In contrast to the effects in the MOLM-13/AZA cells, in the MOLM-13/DAC cells, AZA, but not DAC, induced significant activation of caspase 3/7 and marginally significant activation of caspase 8 and 9.

## 3. Discussion

The resistance of neoplastically transformed cells to therapeutics is the result of several phenotypic changes causing mutual interference, that is ultimately the cause of reduced cellular sensitivity to an antitumor drug. We recently highlighted the possibility of a functional link between ABCB1 transporter expression and an altered cellular response to endoplasmic reticulum stressors that are not substrates for the efflux activity of the pump [46]. In drug-resistant AML cells, in addition to classical markers of resistance (efflux pumps, DNA repair systems, enzymes of the first and second phases of detoxification, etc.), various surface proteins, such as CD33 [47], nestin [48,49], latrophilin [30] and others, also change.

Therefore, it is not surprising that cells overexpressing ABCB1 are also resistant to substances that are not ABCB1 substrates [50]. In the present work, by culturing MOLM-13 cells in medium with stepwise increased concentrations of AZA or DAC for half a year, we obtained cell variants that were resistant to either AZA or DAC without mutual cross-resistance (Figure 1). In addition, these cells did not have substantially changed sensitivity to vincristine, cisplatin, bortezomib and vorinostat. Thus, it can be concluded that the cells obtained generated a phenotype of resistance specifically to either AZA or DAC. Consistently, no considerable differences in ABCB1, ABCC1 and ABCG2 expression were observed (Figure 2).

**Figure 10:**
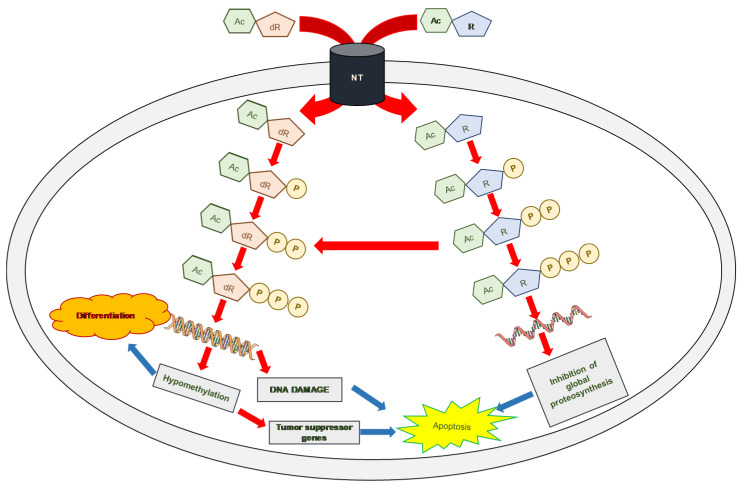
Scheme for gradual phosphorylation of DAC and AZA before they are incorporated into nucleic acids. DAC is incorporated exclusively into DNA, where it causes inhibition of its subsequent methylation and alteration of the gene expression profile. When the promoters of tumor suppressor genes are demethylated, their subsequent expression may initiate controlled cell death processes (most commonly apoptosis). Incorporation of DAC into the DNA structure can result in DNA damage with subsequent initiation of apoptosis (mainly through the intrinsic pathway). In contrast, AZA preferentially incorporates into RNA and causes alterations in the overall metabolism of oligo- and polyconjugates and the subsequent inhibition of global proteosynthesis. Approximately 10–40% of AZA in its nucleotide diphosphate undergoes conversion to the corresponding deoxyribonucleotide diphosphate by ribonucleotide diphosphate reductase and is incorporated into DNA. Symbols: Ac—5-azacytosine; dR—2-deoxyribose; R—ribose; and P—phosphate.

Both AZA and DAC induced cell death with characteristics of apoptosis that could be detected by FAV binding to the outer surface of cells (Figure 3). Both AZA and DAC undergo sequential phosphorylation in cells to the respective nucleotide triphosphates (Figure 10).

Deoxyazacytidine triphosphate is incorporated into DNA, where it induces a strong loss of methylation trace in daughter cells, and thus, induces changes in the phenotype of these cells towards altered differentiation or expression of tumor suppressor genes and apoptosis [51]. However, when incorporated into DNA, it can additionally cause damage to the DNA structure, and thus, activate apoptosis (Figure 10). In contrast, AZA is preferably incorporated into RNA. However, the azacytidine diphosphate formed by sequential phosphorylation can be converted to deoxyazacytidine diphosphate upon ribonucleotide diphosphate reductase activity, which can be incorporated into DNA (Figure 10). For example, in human AML KG-1a cells, the RNA:DNA ratio of AZA incorporation was 65:35 [7]. In the case of incorporation into DNA, the mechanism of action is the same as that described for DAC. When AZA is incorporated into RNA, its synthesis and stability are reduced, leading to disruption of global cellular proteosynthesis [51], and subsequent activation of programmed death mechanisms (most commonly apoptosis) is initiated (Figure 10).

Different mechanisms may be involved in the development of HMA-resistant cell variants, which may lead to cell variants with different phenotypes. Examples of these conditions are the MOLM-13 and SKM-1 cell variants resistant to AZA and P-glycoprotein substrates described in previous work [26,27]. Therefore, during the gradual adaptation of cells to AZA or DAC, the phenotypes of these cells must be carefully checked to determine whether returning to the previous step is necessary.

All these facts indicated that, in our cell models, there was no cross-resistance for the two HMAs, suggesting that when cells become resistant to one cytidine analog, the other can still be used effectively for treatment. This outcome is consistent with the fact that DAC may be effective in patients with MDS previously treated with AZA [52]. In contrast to this finding, Hur et al. [53] described other variants of AZA- and DAC-resistant MOLM-13 cells in which significant cross-resistance was observed. This outcome suggests that the development of the DAC or AZA resistance phenotype in AML cells may not be uniform and is dependent on the protocol used to select/adapt the initially sensitive cells to both HMAs. This outcome is the result of large-scale mechanisms that may be activated in the overall resistance to the respective HMAs, and several of them may lead to resistance against both HMAs.

We detected a decrease in MMP in the MOLM-13 cells after treatment with AZA and DAC, in the MOLM-13/AZA cells after treatment with DAC and in the MOLM-13/DAC cells after treatment with AZA. (Figure 3). It is generally accepted that apoptosis (particularly apoptosis induced through the intrinsic pathway) is associated with depolarization of the MMP. Permeabilization of the mitochondrial membrane and a decrease in MMP seem to play an irreplaceable role in apoptosis progression [54]. During cell death, the formation and activity of the mitochondrial permeability transition pore (mPTP), which causes MMP loss, represents a crucial regulatory feature of the mitochondrial response to cell death stimuli [55]. The aforementioned apoptosis in the MOLM-13 cell variants after treatment with HMAs is linked with typical morphological features that can be detected with forward and side scatter via flow cytometry (Figure 4).

In all three types of MOLM-13 cells, we determined the methylation status of the promoters of genes involved in the intrinsic and extrinsic pathways of apoptosis in the absence and presence of both HMAs (Figure 5). The methylation level was compared with the expression level of the respective genes after estimating the quantity of the respective transcript by qRT-PCR. We found that a decrease in promoter methylation leads to increased expression of the *TNFRSF25* gene, the only gene of 22 detected with a EpiTect^®^ Methyl II Signature PCR Array Kit. This gene showed the highest rate of methylation at CpG promoter sites (92.5%) of the eight genes measured for DNA methylation in urothelial tumor samples from young patients [56]. Additionally, the MOLM-13 cells showed a high degree of methylation of the *TNFRSF25* promoter (98.52%), which was also evident in the MOLM-13/AZA cells but not in the MOLM-13/DAC cells (Figure 3). It appears that in a highly methylated promoter, a small suppression of methylation together with other cellular stimulatory signals can lead to an increase in the expression of the *TNFRSF25* gene. We did not find such a simple relationship between methylation and expression with the other genes. The fact depressed methylation of the respective gene promoter regions did not correlate with the upregulation of these, has already been described for both HMAs [57,58]. In addition, increased expression of the respective genes after AZA or DAC treatment can be explained by a decrease in the methylation of their promoter regions in only a small number of cases. Interesting results were obtained by measuring *GADD45A* transcription and methylation. The expression of this gene and its activity are crucial for the response to genotoxic load and DNA repair after DNA damage [59]. We observed upregulated expression of this gene in the MOLM-13 and MOLM-13/AZA cells after culturing them in the presence of DAC, even when both cell types in this situation had an increased level of methylation of the respective promoter. Thus, it can be stated that *GADD45A* is one of the genes in which the level of promoter methylation may not correlate with its expression level. In contrast, culturing MOLM-13 and MOLM-13/DAC cells in AZA-containing medium resulted in the decrease in *GADD45A* promoter methylation and increased expression. In addition to hypomethylation, the incorporation of HMAs into the structure of DNA also causes damage (Figure 10), which induces the expression of proteins active in DNA repair, including *GADD45A*. Even an increase in the methylation of the *GADD45A* promoter does not appear to be sufficient to stop its induction.

In a set of subsequent experiments, we focused on a more detailed characterization of the type of cell death of the MOLM-13 cell types after HMA treatment. We first determined the expression levels of BCL2 (the best-characterized antiapoptotic protein) and BAX (the best-characterized proapoptotic protein) at the protein level. The results shown in Figure 7 are summarized in Table 3 and provide a comprehensive overview of the findings. The following effects of both HMAs on the expression of BCL2 protein were obtained: DAC caused a decrease in BCL2 protein levels in the MOLM-13 and MOLM-13/AZA cells; similarly, AZA caused a decrease in the expression of this protein in both cell variants but the effect was less pronounced in the MOLM-13/AZA cells. Neither DAC nor AZA induced changes in the expression of the BCL2 protein in the MOLM-13/DAC cell variant. DAC caused an increase in the level of BAX protein in the MOLM-13 and MOLM-13/DAC cells, in which the level of this protein decreased under the influence of AZA. Neither HMAs altered BAX protein levels in the MOLM-13/AZA cells.

The ratio of antiapoptotic to proapoptotic proteins is important for the initiation of the mitochondrial pathway of apoptosis. In the clinic, the BCL2/BAX ratio is used as a predictive marker for estimating the ability of cells to undergo apoptosis after chemotherapy or radiotherapy and thus can be used for estimating the prognosis of treatment [60,61,62]. Therefore, we calculated the BCL2/BAX ratios, and the results are summarized in Table 3. A reduction in the BCL2/BAX ratio (i.e., the predominance of proapoptotic stimulus) was observed for the MOLM-13 and MOLM-13/AZA cells after treatment with both AZA and DAC. In contrast, each HMA induced an increase rather than a decrease in the BCL2/BAX ratio in the MOLM-13/DAC cells. These data suggest that the intrinsic apoptosis pathway may be activated in the MOLM-13 and MOLM-13/AZA cells and inactivated in the MOLM-13/DAC cells after treatment with either HMA.

To verify these results, it is necessary to determine whether the downstream members of the intrinsic apoptotic pathway, which is triggered by the permeabilization of the mitochondrial membrane induced by BAX, i.e., initiating caspase 9 and executive caspase 3 or 7, are activated. We were also interested in whether initiating caspase 8, which is typical of the extrinsic pathway of apoptosis, is also activated. Therefore, we measured the activities of these caspases in the MOLM 13 cells after treatment with HMAs using the Cell MeterTM Multiplexing Caspase 3/7, 8 and 9 activity assay kit (Figure 9). The data shown in Figure 9 are summarized in Table 3. In the MOLM-13 cell types, either HMA caused the activation of caspase 3/7 and caspase 8, without significant activation of caspase 9. This suggests activation of the extrinsic apoptotic pathway. Neither DAC in the MOLM-13/DAC cells nor AZA in MOLM-13/AZA cells induced activation of these caspases. AZA treatment of the MOLM 13/DAC cells induced significant caspase 3/7 activation and only marginally significant caspase 8 and 9 activation (Figure 9). Cell death in this case seems to be the result of a combination of intrinsic and extrinsic apoptotic pathways. DAC in MOLM-13/AZA cells induced significant caspase 3/7 and 8 activation and only marginally significant caspase 9 activation (Figure 9). Therefore, we concluded that in this case, the intrinsic pathway of apoptosis may be involved in cell death, but the extrinsic pathway dominates. The situation is complicated because there are multiple links between the intrinsic and extrinsic apoptotic pathways. For example, initiation of the extrinsic pathway can modulate the intrinsic pathway through the cleavage of BID proteins with activated caspase 8 or caspase 6, which is downstream of mitochondria in the intrinsic pathway and can feed back signals to the extrinsic pathway by cleaving caspase 8 [63]. In addition to these linkages, executive caspase 3 itself can cleave BCL2 [64] and BCL-XL [65] proteins, resulting in feedback for the regulation of the intrinsic apoptosis pathway.

The expression of NF-κΒ regulatory pathway client proteins plays an important role in the fine-tuned regulation of the antiapoptotic/proapoptotic balance and in the prevalence of one of the two (extrinsic or intrinsic) apoptotic pathways [24]. Therefore, we measured the gene expression of canonical and noncanonical NF-κB pathway members (Figure 8). The data shown in Figure 8 are summarized in Table 3. Neither DAC nor AZA changed the gene expression of the *REL* gene (encoding c-Rel protein considered canonical NF-κΒ transcription factor [66]) in the three types of MOLM-13 cells. A slight increase in the expression of the *RELA* gene (encoding the REL-A transcription factor in the canonical NF-κB pathway [66]) was observed in all three types of MOLM-13 cells after AZA treatment (Table 3). DAC treatment of the MOLM-13 and MOLM-13/AZA cells does not induce changes in *RELA* gene expression, and in the MOLM-13/DAC, *RELA* expression was slightly reduced. The gene expression of the second member of the transcription factor dimer in the canonical NF-κB pathway, *NFKB1* [66], was reduced in the MOLM-13 and MOLM-13/AZA cells after treatment with DAC or AZA (Table 3). AZA at a concentration of 0.5 μM induced an increase in the expression of this transcription factor in the MOLM-13/DAC cells, but this increase was not observed at a concentration of 1.0 μM. There was no change in *RELA* expression under the influence of DAC in these cells (Table 3). A slight increase in the expression of the *RELB* gene encoding the REL-B transcription factor of the noncanonical NF-κB pathway [66] was observed in the MOLM-13 cells after treatment with either HMA and in the MOLM-13/AZA cells after treatment with DAC. AZA caused a more pronounced upregulation of this gene in the MOLM-13/DAC cells (Table 3). Neither DAC in the MOLM-13/DAC cells nor AZA in the MOLM-13/AZA cells was able to alter *RELB* gene expression. Both HMAs caused a decrease in the expression of the *NFKB2* gene encoding the second member of the transcriptional dimer in the noncanonical NF-κB pathway [66] in the MOLM-13 cells. AZA (at concentrations of 0.5 and 1.0 μM) caused a slight increase in the expression of the *NFKB2* gene in the MOLM-13/DAC cells, but it only had an effect in the MOLM-13/AZA at a concentration of 0.5 μM (Table 3). However, AZA at a concentration of 1.0 μM resulted in a five-fold increase in the expression of this gene. Neither DAC in the MOLM-13/DAC cells nor AZA in MOLM-13/AZA cell variants induced a change in the expression of the *NFKB2* gene. These data suggest that the NF-κB pathway may be involved in the regulation of HMA-induced apoptosis in the MOLM-13 cell line and its resistant variant sublines. The results indicate that switching between canonical and noncanonical pathways may be induced by HMAs. However, the regulatory mechanisms of the NF-κB pathway appear to be very complex, and further targeted research will be needed to accurately understand the causal relationships.

## 4. Materials and Methods

### 4.1. Cell Culture Conditions

The MOLM-13 cell line (ACC 554), derived from the peripheral blood of a 20-year-old patient with AML developed from myelodysplastic syndromes (supplied by Leibniz-Institute DSMZ—Deutsche Samsung von Microorganism und Zellkulturen GmbH, Braunschweig, Germany), was used in this study. The sensitive MOLM-13 cell line was adapted to 5-aza-2′-deoxycytidine (DAC) and azacytidine (AZA) (both from Sigma Aldrich, St. Louis, MO, USA) over a 6-month period with repeated passaging in medium containing stepwise increases in drug concentrations beginning at 0.1 nmol/L. This procedure yielded DAC-resistant MOLM-13/DAC and AZA-resistant MOLM-13/AZA cell variants. The cell lines were cultured in 5 mL of RPMI medium (5 × 10^5^ cells) containing 12% fetal bovine serum (both from Gibco, Langley, OK, USA), 100,000 units/l penicillin and 50 mg/L streptomycin (both from Sigma Aldrich, St. Louis, MO, USA) for one or two days at 37 °C in a humidified atmosphere containing 5% CO_2_.

In a previous study, we prepared MOLM-13 cell variants resistant to AZA and Pgp substrates that overexpressed P-glycoprotein [26,27]. In the current study, we aimed to prepare cell variants resistant to either AZA or DAC with a mechanism of resistance specifically focused on these HMAs. Therefore, during the whole procedure of cell variant preparation, we controlled the expression of ABC transporters, and cells with improved ABC transporter expression were excluded.

### 4.2. Cell Viability MTS Assay

Sensitive and resistant MOLM-13 cell variants were incubated under standard culture conditions with different concentrations of BTZ, cisPt, VCR or SAHA for 48 h and various concentrations of AZA or DAC for 72 h. The cell lines were treated with AZA/DAC every 24 h. After cultivation, the CellTiter 96^®^ AQueous one solution cell proliferation assay (MTS assay) (Promega, Madison, WI, USA) was used to determine the metabolic activity of cells according to the manufacturer’s protocol. The IC50 was computed by nonlinear regression according to Equation 1 using SigmaPlot for Windows version 8.02. The data represent computed values ± standard error with 30 degrees of freedom.

### 4.3. Genomic DNA Isolation from Cell Lines

MOLM-13, MOLM-13/DAC and MOLM-13/AZA cells were collected after 72 h of cultivation with 1 μmol/L AZA or DAC. The cells were treated with AZA/DAC every 24 h. Genomic DNA was extracted using a commercially available Allprep DNA/RNA Kit (Qiagen, Hilden, Germany) according to the manufacturer’s instructions. The DNA purity and concentration were estimated by measuring the absorbance at 260 nm with a nanophotometer (Implen, Munich, Germany).

### 4.4. DNA Methylation Detection on Human Apoptosis Genes

To detect the promoter methylation status of 22 genes associated with apoptosis, we used a unique methylation platform, an EpiTect^®^ Methyl II Signature PCR Array Kit (Qiagen, Hilden, Germany). This method is based on the detection of remaining input DNA after cleavage with a methylation-sensitive and/or a methylation-dependent restriction enzyme using real-time PCR. We performed restriction digestion using an EpiTect^®^ Methyl II DNA Restriction Kit (Qiagen, Hilden, Germany) following the manufacturer’s protocol. The methylation status of the gene promoter regions (the relative amount of methylated and unmethylated DNA fractions) was calculated with the analysis program provided by the manufacturer (Qiagen, Hilden, Germany) using the ΔCt method.

### 4.5. Detection of BCL2, BAX and GAPDH Protein Levels in MOLM-13, MOLM-13/DAC, and MOLM 13/AZA Cells

Sensitive and resistant MOLM-13 cell variants were cultured in standard medium for 24, 48 and 72 h with or without AZA/DAC (0.5 and 1 μmol/L). The cells were treated with AZA/DAC every 24 h. After incubation, the cells were harvested, and whole-cell lysates were prepared by homogenization in SoluLyse (Sigma-Aldrich, St. Louis, MO, USA) according to the manufacturer’s instructions. Briefly, the cells were washed twice with PBS and centrifuged (2800 rpm, 5 min), and the pellets were extracted with SoluLyse buffer for 15 min while shaking. The samples were centrifuged (3000 g, 15 min), and the supernatants were stored for further analysis. In addition, the protein concentration was determined using a Lowry assay. Sample proteins were separated by sodium dodecyl sulfate-polyacrylamide electrophoresis (SDS–PAGE) in a 12% gel. The proteins were then transferred by electroblotting to a nitrocellulose membrane (GE Healthcare Europe GmbH, Vienna, Austria). Rabbit antibodies directed against BCL2 (SC-492), BAX (SC-493) and GAPDH (MAB374), as an internal control (Santa Cruz Biotechnology, Dallas, TX, USA), were used as primary antibodies, and a goat anti-rabbit antibody (SC-2054) conjugated with horseradish peroxidase (Santa Cruz Biotechnology, Dallas, TX, USA) served as a secondary antibody. Protein bands were visualized by ECL detection (GE Healthcare Europe GmbH, Vienna, Austria) and Amersham Imager 600 (GE Healthcare Europe GmbH, Pittsburgh, PA, USA). Protein quantities were established by densitometry using ImageQuant software (GE Healthcare Europe GmbH, Pittsburgh, PA, USA), and are expressed relative to GAPDH.

### 4.6. Detection of AZA- and DAC-Induced Apoptosis and Necrosis in Sensitive and Resistant MOLM-13 Cells

Cells (1 × 10^6^ cells/mL) were incubated for 24 h, 48 h and 72 h with 0, 0.5, 1, 2, or 5 μmol/l AZA or DAC under standard culture conditions, and the cell lines were treated with AZA/DAC every 24 h. After this incubation period, the proportion of apoptotic and necrotic cells was measured using an annexin V (Roche, Mannheim, Germany)/propidium iodide kit (Calbiochem, San Diego, CA, USA). The cells were washed twice with PBS and gently resuspended in binding buffer containing 0.5 μg/mL FITC-labeled annexin V. The mixtures were incubated for 15 min at room temperature in the dark and then centrifuged (2500 rpm, 15 min). The resulting sediments were resuspended in binding buffer, and propidium iodide (final concentration of 0.6 μg/mL) was added to each sample, after which the samples were analyzed by flow cytometry using an Accuri C6 flow cytometer (BD Bioscience, San Jose, CA, USA).

### 4.7. Determination of the REL, NFKB1, NFKB2, RELA, and RELB Transcript Levels in MOLM-13 and MOLM-13/DAC and MOLM-13/AZA Cells

MOLM-13, MOLM-13/DAC and MOLM-13/AZA cells were collected after 72 h of cultivation, and total RNA was isolated using TRI Reagent (MRC, Cincinnati, OH, USA) according to the manufacturer’s instructions. Reverse transcription (RT) was performed with 2 μg of DNase I (from Thermo Fisher Scientific, Waltham, MA, USA)-treated RNA using a RevertAid™ H Minus First-Strand cDNA synthesis kit (Thermo Fisher Scientific, Waltham, MA, USA) according to the manufacturer’s protocol. PCR was performed in a total volume of 25 μL using a PCR kit according to the manufacturer’s protocol (Thermo Fisher Scientific, Waltham, MA, USA). After heating the samples at 94 °C for 3 min to inactivate the reverse transcriptase, the samples were subjected to 30 cycles of denaturation (95 °C, 30 s), annealing (59 °C, 30 s for *NFKB1*; 57.4 °C, 30 s for *NFKB2*; 56.3 °C, 30 s for *RELA*; 58 °C, 30 s for *REL*; 58 °C, 30 s for *RELB*; 54.4 °C, 30 s for *ACTB*; 57 °C, 30 s for *ABCB1*; 57.3 °C, 30 s for *ABCC1*; 59.1 °C, 30 s for *ABCG2*), extension (72 °C, 90 s), and a final extension (72 °C for 10 min). The primer sequences were: *ACTB*—5′-CTG GGA CGA CAT GGA GAA AA-3′ and 5′-AAG GAA GGC TGG AAG AGT GC-3′, which produce a 564 bp product; *ABCB1*—5′-AAG TTG TAT ATG GTG GGA ACT-3′ and 5′- AAT TTT GTC ACC AAT TCC TTC ATT-3′, which produce a 429 bp product; *ABCC1*—5′-AGA AGT CTG GAC GTC CCT G-3′ and 5′-ACA CCA AGC CGG CG TCT TT-3′, which produce a 404 bp product; *ABCG2*—5′-TCT GGA GAT GTT CTG ATA AAT GGA-3′ and 5′-GAC CTA ACT CTT GAA TGA CCC TGT-3′, which produce a 202 bp product; *NFKB1*—5′-ACT AGC ACA AGG AGA CAT GAA ACA-3′ and 5′-TTT TGT TGA GAG TTA GCA GTG AGG-3′, which produce a 455 bp product; *NFKB2*—5′-GAA CAG CCT TGC ATC TAG CC-3′ and 5′-TTT TCA GCA TGG ATG TCA GC-3′, which produce a 190 bp product *RELA*—5′-TCT GCT TCC AGG TGA CAG-3′ and 5′-GCC AGA GTT TCG GTT CAC-3′, which produce a 137 bp product; *RELB*—5′-CTG CTT CCA GGC CTC ATA TC-3′ and 5′- CGC AGC TCT GAT GTG TTT GT-3′, which produce a 108 bp product; *REL*—5′-CGA ACC CAA TTT ATG ACA AC-3′ and 5′-TTT TGT TTC TTT GCT TTA TTG C-3′, which produce a 370 bp product. The PCR products were separated on a 1.7% agarose gel (Lonza, Rockland, ME, USA), and the gel was visualized with GelRed™ nucleic acid gel stain (Biotium, Fremont, CA, USA) using an Amersham Imager 600 (GE Healthcare Europe GmbH, Pittsburgh, PA, USA). The mRNA quantities were established by conducting a densitometric evaluation with ImageQuant™ image analysis software (GE Healthcare Europe GmbH, Pittsburgh, PA, USA). All reactions were performed in triplicate, and the mRNA levels were normalized to the level of *ACTB*.

### 4.8. Measurement of JC-1 Fluorescence in Sensitive and Resistant MOLM-13 Cells by Fluorescence Cytometry

Cells (2 × 10^5^ cells/mL) were incubated for 72 h with 0 or 1 μmol/L AZA or DAC under standard culture conditions, while cell lines were treated with AZA/DAC every 24 h. After culturing, the cells were harvested by centrifugation (5 min at 500× *g*) and then resuspended in 200 µL of RPMI medium without fetal bovine serum. JC-1 (Sigma Aldrich, St. Louis, MO, USA) was added to a final concentration of 2.5 µM, and the cells were shaken in the dark at 37 °C for 15 min. CCCP was used as a prototypical mitochondrial uncoupler. Finally, the cells were counted in a BD Accuri C6 flow cytometer (BD Bioscience, San Jose, CA, USA) according to the manufacturer’s instructions [32].

### 4.9. qRT-PCR

MOLM-13, MOLM-13/DAC and MOLM-13/AZA cells were collected after 72 h of cultivation, and total RNA was isolated using TRI Reagent (MRC, Cincinnati, OH, USA) according to the manufacturer’s instructions and subjected to reverse transcription with a SuperScript VILO cDNA synthesis kit (Thermo Fisher Scientific, Waltham, MA, USA) according to the manufacturer’s protocol. qRT-PCRs were performed in 96-well plates with a CFX96 Real-Time System C1000 Touch Thermal Cycler (Bio-Rad Europe GmbH, Basel, Switzerland). The reactions were carried out in a final volume of 20 µL consisting of 10 µL of TaqMan Gene Expression Master Mix (Thermo Fisher Scientific, Waltham, MA, USA), 1 µL of TaqMan Gene Expression Assay—Hs01060665_g1 (for *ACTB*), Hs00708019_s1 (for *BCL2L11*), Hs03004661_g1 (for *BCLAF*), Hs00609632_m1 (for *BID*), Hs00154189_m1 (for *BIK*), Hs00234387_m1 (for *CASP3*), Hs00234480_m1 (for *DAPK1*), Hs00169255_m1 (for *GADD45A*), and Hs00600930_g1 (for *TNFRSF25*) (Thermo Fisher Scientific, Waltham, MA, USA)—and 9 µl of cDNA diluted with DNase/RNase-free water to a final concentration of 100 ng/reaction. All reactions were performed in triplicate, and mRNA quantities were normalized to the quantity of *ACTB*. The relative expression of the target genes was calculated using the 2^−ΔΔCt^ method [67].

### 4.10. Detection of Caspase Activity

Cells (2 × 10^5^ cells/mL) were incubated for 72 h with 0, 0.5, 1.0 μmol/L AZA or DAC under standard culture conditions, while the cell lines were treated with AZA/DAC every 24 h. After incubation, 1.5 × 10^5^ cells were harvested, resuspended in fresh medium and transferred to 96-well plates (100 µL/cell). The caspase enzymatic activity was measured using a Cell MeterTM Multiplexing Caspase 3/7, 8 and 9 Activity Assay Kit (AAT Bioquest, Sunnyvale, CA, USA) according to the manufacturer’s protocol [45]. The florescence intensity in each well was measured using a 96-well fluorescence reader FLx800 (Bio-tek).

### 4.11. Statistical Analysis and Data Processing

Numerical data are expressed as the mean ± SD of three independent measurements. Statistical significance was assessed by unpaired Student’s t-test using SigmaPlot 8.0 software (Systat Software, Inc., San Jose, CA, USA) or by one-way ANOVA.

## 5. Conclusions

Variants of the human MOLM-13 AML cell line MOLM-13/DAC and MOLM-13/AZA prepared in this research are resistant to DAC and AZA, respectively. No cross-resistance to the opposite cytidine derivative of these cell variants was found. When cell death was induced by HMAs, the cells underwent apoptosis. In parental MOLM-13 cells, the extrinsic apoptosis pathway was activated after treatment with both HMAs. AZA in MOLM-13/DAC and DAC in MOLM-13/AZA induced apoptosis through the combination of the extrinsic and intrinsic pathways. Each cell variant represents a suitable cell model for characterizing the differences in the distribution of resistance between two HMAs.

## Figures and Tables

**Table 1:** Genes for which methylation was detected by the EpiTect^®^ Methyl II Signature PCR Array Kit.

Gene No.	Gene Abbreviation	Gene Description	Gene No.	Gene abbreviation	Gene Description
**Intrinsic apoptotic pathway**			12	*CIDEB*	Cell death-inducing DFFA-like effector B
1	*APAF1*	Apoptotic peptidase-activating factor 1	13	*DFFA*	DNA fragmentation factor, 45 kDa, alpha polypeptide
2	*BAD*	BCL2-associated agonist of cell death	14	*GADD45A*	Growth arrest and DNA damage-inducible, alpha factor
3	*BAX*	BCL2-associated X protein	15	*HRK*	Harakiri, BCL2-interacting protein
4	*BCL2L11*	BCL2-Iike 11	16	*TP53*	Tumor protein p53
5	*BCLAF1*	BCL2-associated transcription factor	**Extrinsic apoptotic pathway**		
6	*BID*	BH3-interacting domain death agonist	17	*CRADD*	CASP2- and RIPK1-domain containing adaptor with death domain
7	*BIK*	BCL2-interacting killer	18	*DAPK1*	Death-associated protein kinase 1
8	*BIRC2*	Baculoviral IAP repeat-containing 2	19	*FADD*	Fas(TNFRSF6)-associated via death domain
9	*BNIP3L*	BCL2-interacting protein 3-like	20	*LTBR*	Lymphotoxin beta receptor (TNFR superfamily, member 3)
10	*CASP3*	Caspase3, apoptosis-related cysteine peptidase	21	*TNFRSF21*	Tumor necrosis tactor receptor superfamily, member 21
11	*CASP9*	Caspase 9, apoptosis-related cysteine peptidase	22	*TNFRSF25*	Tumor necrosis tactor receptor superfamily, member 25

**Table 2:** Activity of the caspases in the parental and two resistant variants of MOLM-13 cells.

	MOLM-13 ^a^	MOLM-13/DAC ^b^	MOLM-13/AZA ^c^	a vs. b *p*	a vs. c *p*
	(Activity in MOLM-13 Was Taken as 100%)				
Caspase 3/7	100.0 ± 9.9	96.9 ± 3.4	89.8 ± 3.5	0.78	0.39
Caspase 8	100.0 ± 2.0	103.8 ± 4.3	101.1 ± 3.8	0.47	0.80
Caspase 9	100.0 ± 7.4	90.3 ± 2.6	105.7 ± 6.9	0.28	0.60

*p* = probability of a significant difference.

**Table 3:** Summary of the expression/activity of proteins active in apoptosis.

Cells	MOLM-13		MOLM-13/DAC		MOLM-13/AZA	
Treatment	DAC	AZA	DAC	AZA	DAC	AZA
Gene/Protein	Antiapoptotic BCL2 and proapoptotic BAX proteins					
**BCL2 protein**	**↓**	**↓**	**0**	**0**	**↓**	_**↓**_
**BAX protein**	**↑**	**↓**	**↑**	**↓**	**0**	**0**
**BCL2/BAX ratio**	**↓**	**0**/↓ *	**↑**/0 *	**↑**	**↓**	**↓**
	**Caspases**					
***CASP 3*** **mRNA**	**↓**	**_↑_**	**↓**	**↑**	**↑**	**0**
**Casp-3/7 act.**	**↑**	**↑**	**0**	**↑**	**↑**	**0**
**Casp-8 act.**	**↑**	**↑**	**0**	**_↑_**	**↑**	**0**
**Casp-9 act.**	**0**	**0**	**0**	**_↑_**	**_↑_**	**0**
	**NF-κB**					
***REL*** **mRNA**	**0**	**0**	**0**	**0**	**0**	**0**
***RELA*** **mRNA**	**0**	**_↑_**/0 *	**0**	**_↑_**	_**↓**_	**_↑_**
***NFKB1*** **mRNA**	**↓**	**↓**	**0**	**↑**/0 *	**↓**	**↓**
***RELB*** **mRNA**	**_↑_**	**_↑_**	**0**	**↑**	**_↑_**	**0**
***NFKB2*** **mRNA**	**↓**	**↓**	**0**	**_↑_**	**0**	**_↑_**/↑ *

**↑** and **_↑_** = increasingly pronounced upregulation; **↓** and **_↓_** = increasingly pronounced downregulation. **0** = HMAs did not induce any effect. * = first and second symbols are valid for HMA concentrations of 0.5 and 1.0 μM, respectively.

## Data Availability

Additional data as well as resistant variants of MOLM-13 cells are available from the authors.

## Supplementary Materials

Figures S1–S7 are available online at https://www.mdpi.com/1422-0067/22/4/2076/s1.

## Abbreviations

ABC	ATP binding cassette
ABCB1 (C1, G2)	ABC transporter B1 (C1, G2)
*ACTB*	Gene for β-actin
AML	Acute myeloid leukemia
*APAF1*	Apoptotic peptidase activating factor 1
AZA	5-Azacytidine
*BAD*	BCL2-associated agonist of cell death
BAX	BCL2-associated X protein
BCL2	B-Cell CLL/Lymphoma 2
*BCL2L11*	BCL2-like 11
*BCLAF1*	BCL2-associated transcription factor
*BID*	BH3 interacting domain death agonist
*BIK*	BCL2-interacting killer
*BIRC*	Baculoviral IAP repeat-containing 2
BM	Bone marrow
*BNIPL3*	BCL2-interacting protein 3-like
BTZ	Bortezomib
*CASP3*	Caspase 3
*CASP9*	Caspase 9
CCCP	Carbonyl cyanide m-chlorophenylhydrazone
CD33	Cluster of differentiation 33 protein
*CIDEB*	Cell death- inducing DFFA like effector B
cisPt	Cisplatin
DAC	5-Aza-2′-deoxycytidine
*DFFA*	DNA fragmentation factor, 45 kDa, alpha polypeptide
DNA	Deoxyribonucleic acid
DNaseI	Deoxyribonuclease I
*DR3*	Death receptor 3
FADD	Fas (TNFRSF6)-associated via death domain
FAV	FITC-linked annexin V
FITC	Fluorescein isothiocyanate
*GADD45A*	Growth arrest and DNA damage-inducible, alpha factor
GAPDH	Glyceraldehyde 3-phosphate dehydrogenase
*GRADO*	CASP2 and RIPK1 domain containing adaptor with death domain
HMA	Hypomethylating agents
HMC-1,2 cells	Human mast cell leukemia cell line
*HRK*	Harakiri, BCL2 Interacting protein
JC-1	5,5′,6,6′-tetrachloro-1,1′,3,3′-tetraethyl-imidacarbocyanine iodide
*LTBR*	Lymphotoxin beta receptor (TNFR superfamily, member 3)
MDS	Myelodysplastic syndromes
MMP	Mitochondrial membrane potential
MOLM-13/AZA	AZA resistant MOLM-13 cells
MOLM-13/DAC	DAC resistant MOLM-13 cells
mPTP	Mitochondrial permeability transition pore
mRNA	Messenger RNA
*NFKB1* *(2)*	Nuclear Factor-Kappa B Subunit 1 (2)
NF-κB	Nuclear Factor-Kappa B
PCR	Polymerase chain reaction
Pgp	P glycoprotein
PI	Propidium iodide
qRT-PCR	Quantitative reverse transcription—PCR
*REL*	V-Rel Avian Reticuloendotheliosis Viral Oncogene Homolog
*RELA*	V-Rel Avian Reticuloendotheliosis Viral Oncogene Homolog A
*RELB*	V-Rel Avian Reticuloendotheliosis Viral Oncogene Homolog B
RNA	Ribonucleic acid
RT-PCR	Reverse transcription—PCR
SAHA	Suberanilohydroxamic acid
TNFR	Tumor necrosis factor receptor
*TNFRSF21 (25)*	Tumor necrosis factor receptor superfamily 21 (25)
*TP53*	Tumor protein p53
TRADD	TNFR-associated death domain
