# Supplementary material for: Changes in Apoptotic Pathways in MOLM-13 Cell Lines after Induction of Resistance to Hypomethylating Agents
Source: Int J Mol Sci. 2021 Feb 19;22(4):2076. doi: 10.3390/ijms22042076 (PMC7923013; doi:10.3390/ijms22042076)
Supplement: Supplementary file 1 [file ijms-22-02076-s001.pdf]

International Journal of *Molecular Sciences*

MDPI

Supplementary files

Janotka et al., "Changes in Apoptotic Pathways in MOLM-13 Cell Lines after Induction of Resistance to Hypomethylating Agents."

1. Supplementary results

1.1 Effect of AZA and DAC on apoptosis/necrosis progression in the sensitive MOLM-13 cell line and the resistant variant MOLM-13/AZA and MOLM-13/DAC cell sublines.

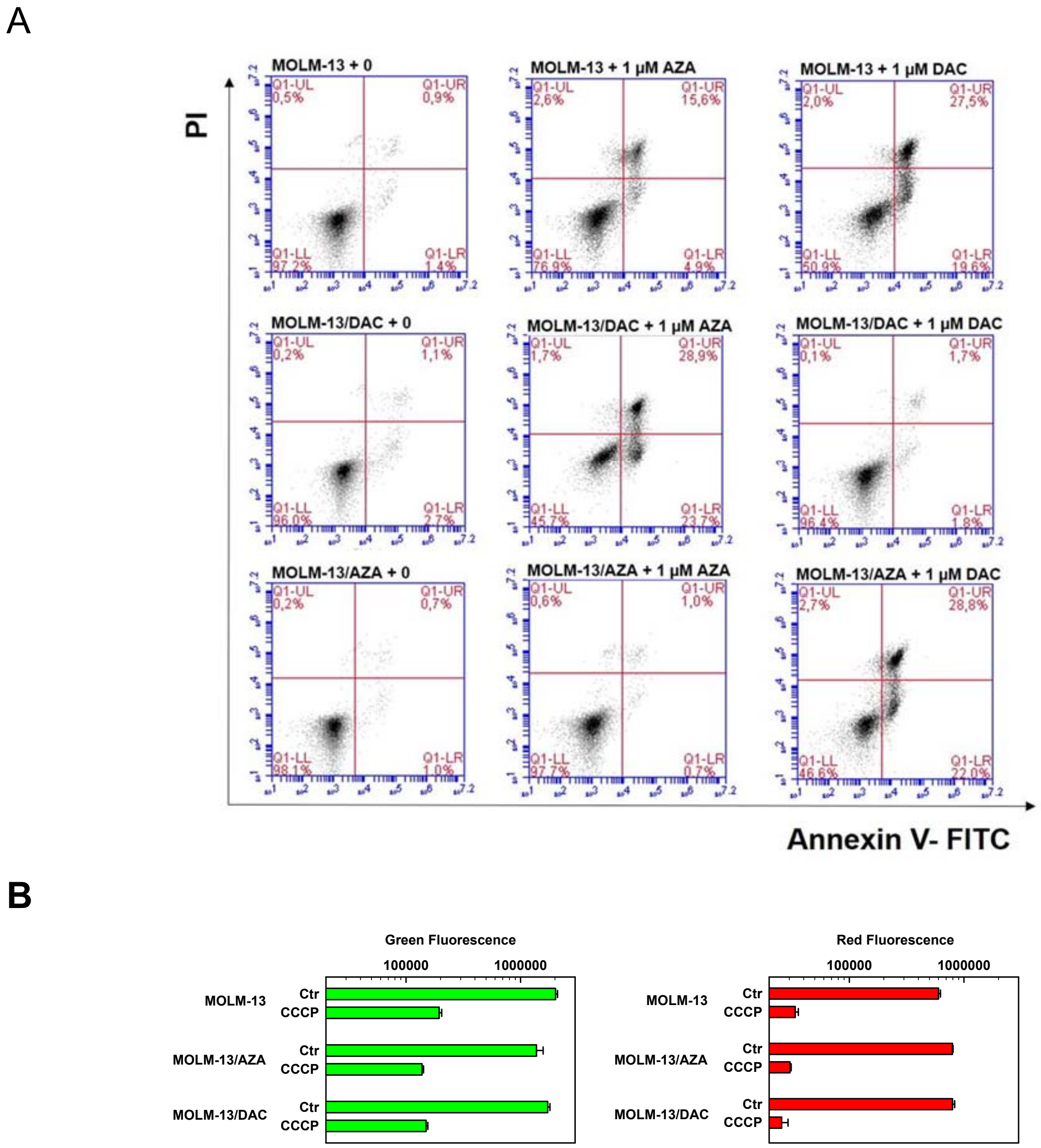

Figure S1: Panel A: Results of the annexin-V/propidium iodide assay of apoptosis/necrosis. Cells were incubated for 72 hours in growth medium in the absence (+0) or presence of 1 µM AZA or DAC in a CO_2 incubator. The cells were treated with AZA/DAC every 24 hours. After this period, apoptosis/necrosis progression was assayed using an annexin-V/propidium iodide kit. Specific cell fluorescence was measured by fluorescence flow cytometry. Representative plots of three independent measurements are shown. Panel B: JC-1 green and red fluorescence detected by flow cytometry in the absence (Ctr) and presence of carbonyl cyanide m-chlorophenyl hydrazone (CCCP) at 50 µM.

1.2 Side and forward scatter of viable MOLM-13, MOLM-13/AZA and MOLM-13/DAC cells after cultivation with AZA and DAC.

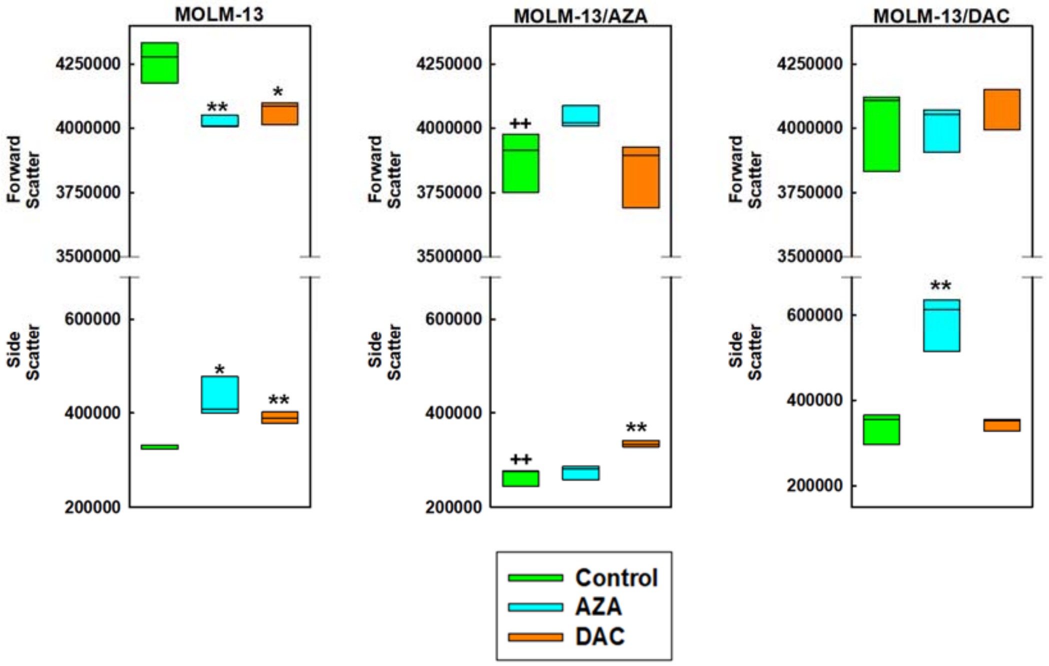

Figure S2: Box plots showing the forward and side scatter of viable MOLM-13, MOLM-13/DAC, MOLM-13/AZA cells cultivated in the presence or absence of AZA or DAC (1 μM). Only cells that were not labeled by FAV or PI were evaluated (Figure 3). Boxes were constructed by SigmaPlot for Windows (Version 8.02, Systat Software GmbH, Erkrath, Germany) based on three independent measurements. Statistical significance: ++ — values differ from the data obtained for the MOLM-13 cells at the level $p<0.01$; * — values differ from the data in the absence of AZA or DAC at the level $p<0.05$; ** — values differ from the data in the absence of AZA or DAC at the level $p<0.01$. The top and bottom of the box represent the 5th and 95th percentiles, and the horizontal line in the box represents the median value.

1.3 Measurement of the relative expression and methylation of CASP3, DAPK1, BCL2L11, BCLAF1, BID, and BIK in the sensitive and resistant cell lines.

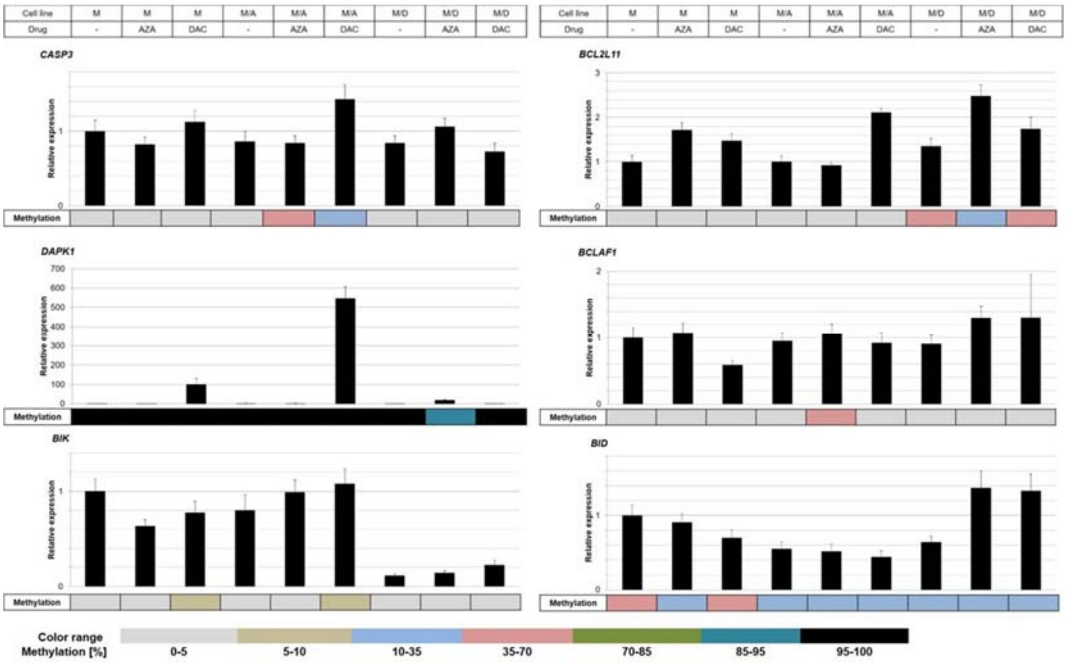

Figure S3: Changes in the relative expression and methylation of *CASP3*, *DAPK1*, *BCL2L11*, *BCLAF1*, *BID*, and *BIK* in the sensitive and resistant cell lines. M — MOLM-13-sensitive cell line; M/A — MOLM-13/AZA, AZA-resistant subline; and M/D — MOLM-13/DAC, DAC-resistant subline. The cells were incubated for 72 hours in growth medium in the absence or presence of AZA (1 μM) or DAC (1 μM) in a CO_2 incubator. The cells were treated every 24 hours with either AZA or DAC. *ACTB* was used as an internal control. The data are expressed as the mean ± SD of three independent measurements.

1.4 *Changes in the protein levels of BCL2 and BAX in the MOLM-13, MOLM-13/DAC, and MOLM-13/AZA cells after 24 and 4 weeks in the presence of DAC or AZA*

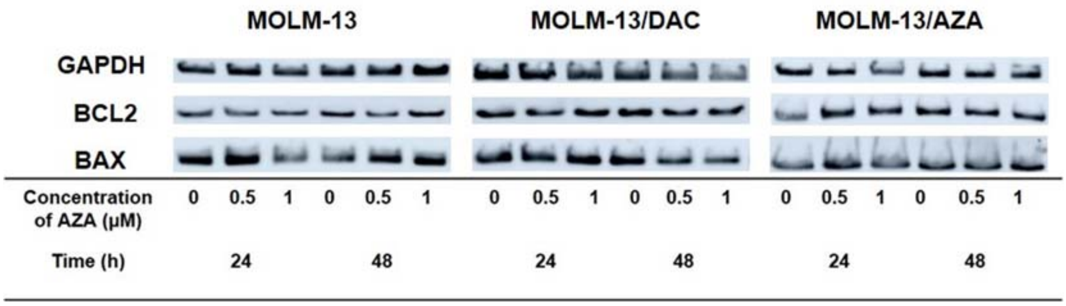

Figure S4: Changes in the levels of BCL2 and BAX proteins in the sensitive and resistant cell lines as determined by Western blot analysis. The cells were incubated for 24 and 48 hours in growth medium in the absence or presence of AZA in a CO_2 incubator. The cells were treated every 24 hours with AZA. The level of GAPDH protein was used as an internal control.

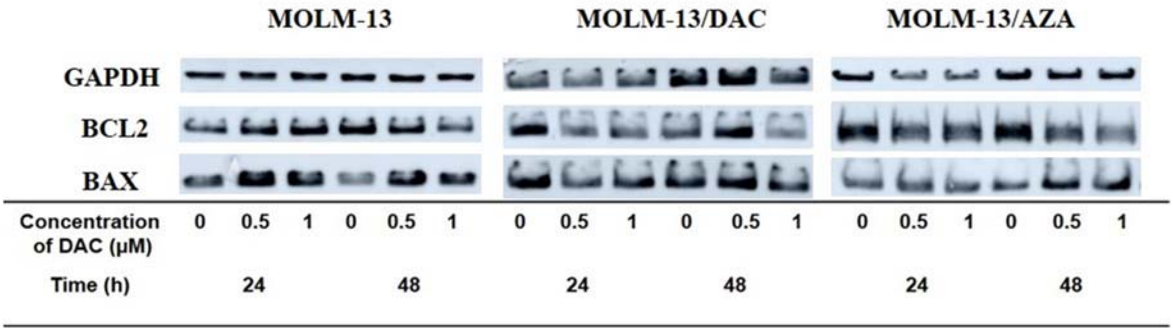

Figure S5: Changes in the levels of BCL2 and BAX proteins in the sensitive and resistant cell lines as determined by Western blot analysis. The cells were incubated for 24 and 48 hours in growth medium in the absence or presence of DAC in a CO_2 incubator. The cells were treated every 24 hours with DAC. The level of GAPDH protein was used as an internal control.

1.5 Expression of the REL, RELA, RELB, NFKB1 and NFKB2 genes in the sensitive and resistant cell lines

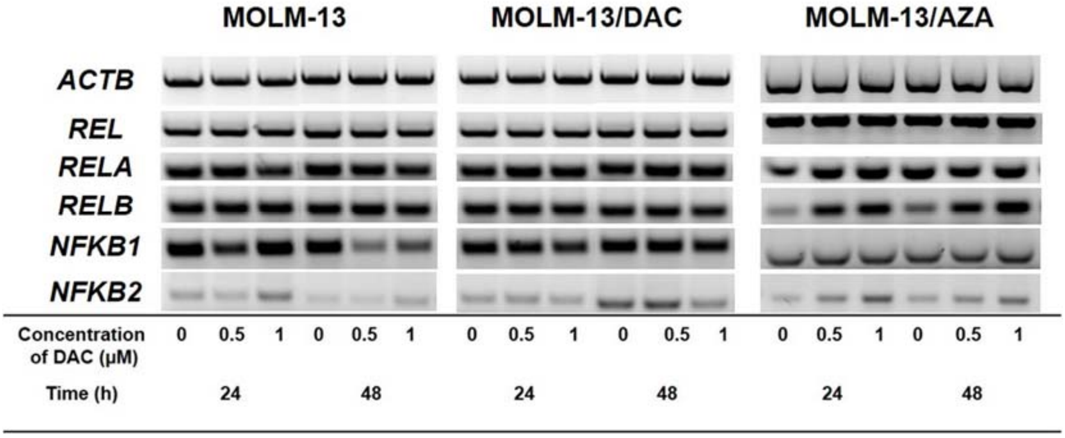

Figure S6: Changes in the relative expression of the *REL, RELA, RELB, NFKB1* and *NFKB2* genes in the sensitive and resistant cell lines as determined by RT-PCR. The cells were incubated for 24 or 48 hours in growth medium in the absence or presence of DAC in a CO_2 incubator. The cells were treated every 24 hours with DAC. *ACTB* was used as an internal control.

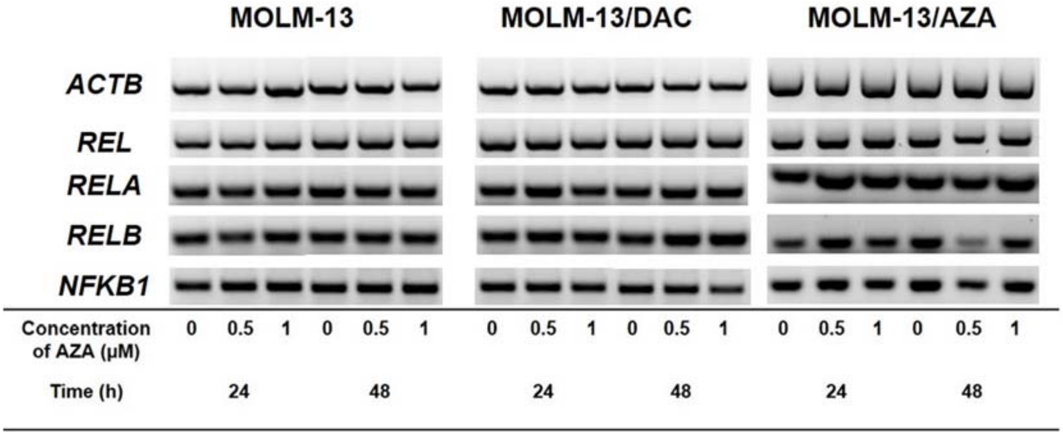

Figure S7: Changes in the relative expression of the *REL, RELA, RELB, NFKB1* and *NFKB2* genes in the sensitive and resistant cell lines as determined by RT-PCR. The cells were incubated for 24 or 48 hours in growth medium in the absence or presence of AZA in a CO_2 incubator. the Cells were treated every 24 hours with AZA. *ACTB* was used as an internal control.
